# Vocal Fold Granuloma Causing Acute Airway Obstruction in a Pediatric Patient

**DOI:** 10.7759/cureus.115794

**Published:** 2026-09-04

**Authors:** Brittani P Kongala, Maaz S Haji, Monique Martin, Francesca C Viola, Michele M Carr

**Affiliations:** 1 College of Medicine, Florida State University College of Medicine, Tallahassee, USA; 2 College of Medicine, Chicago Medical School, Rosalind Franklin University of Medicine and Science, Chicago, USA; 3 College of Medicine, Jacobs School of Medicine and Biomedical Sciences, University at Buffalo, Buffalo, USA; 4 Otolaryngology - Head and Neck Surgery, Jacobs School of Medicine and Biomedical Sciences, University at Buffalo, Buffalo, USA

**Keywords:** airway obstruction, intubation, laryngeal granuloma, pediatric, vocal fold granuloma

## Abstract

Vocal fold granuloma (VFG) is a benign laryngeal lesion caused by mucosal trauma to the arytenoid cartilage. Pediatric VFG is rare and is most often associated with prolonged or traumatic intubation. We present the case of a seven-year-old male who developed a 1-cm pedunculated VFG following two separate brief intubations for minor procedures. The lesion produced a dynamic “ball-valve” effect, resulting in biphasic stridor, breathy dysphonia, and intermittent airway obstruction. While computed tomography (CT) imaging was inconclusive, laryngoscopy identified a pedunculated lesion prolapsing into the subglottis with near-complete intermittent glottic obstruction. The mass was successfully excised, and the patient was extubated after a short course of intravenous steroids with complete resolution of symptoms. This case emphasizes that VFG should remain in the differential diagnosis for pediatric patients presenting with stridor and airway compromise following even brief periods of intubation.

## Introduction

Vocal fold granuloma (VFG), also known as vocal process granuloma, is a benign lesion of the larynx that most often develops secondary to abnormal wound healing. These lesions are most commonly associated with irritation or trauma to the larynx, especially the medial aspect of the vocal process of the arytenoid cartilage [1,2]. In children, intubation-related laryngeal injury spans a wide spectrum from mild edema and dysphonia to granulation tissue formation, stenosis, and impaired vocal fold mobility [3]. However, VFGs by themselves appear to be uncommon in the pediatric population [3-5]. Some reported risk factors include prolonged or traumatic intubation, laryngopharyngeal reflux disease, and infection [4,6]. Granulomas have been reported after relatively brief periods of intubation, including durations of less than one hour [4,6].

VFG has a variable clinical presentation, which can make the diagnosis challenging. Patients may present with dysphonia, stridor, or dyspnea after a prior intubation. Symptoms may appear days to months later rather than immediately post-extubation [3,4]. VFG morphology can vary from case to case, but VFGs are usually either sessile, ulcerated, or pedunculated lesions. In rare instances, pedunculated VFGs can behave like a dynamic “ball-valve” lesion that intermittently prolapses into the glottis, causing episodic airway obstruction [7,8]. This mobility is clinically important because even though symptoms may fluctuate, the lesion poses a substantial airway risk. This is especially true with larger lesions.

Consequently, management of this condition depends on the type of presentation. Conservative management is often utilized for nonobstructive lesions, while surgery is reserved for refractory symptoms, significant voice impairment, or imminent airway compromise [9,10]. We present a child with a pedunculated VFG causing intermittent airway obstruction after two prior brief intubations.

## Case presentation

A seven-year-old male presented to the Emergency Department (ED) with dyspnea, breathy voice, dysphonia, hoarseness, and biphasic stridor. These symptoms were episodic in nature and had been progressively worsening over the past several months. His past medical history included two brief intubations within the past six months for two separate procedures, one dental and one urologic. Plain radiographs taken in the ED suggested a round mass in the subglottis (Figure 1). Subsequent computed tomography (CT) of the neck was normal and did not reveal any subglottic mass (Figure 2). However, bedside flexible fiberoptic laryngoscopy revealed a 1-cm, smooth, pink, pedunculated lesion arising from the right vocal fold. During respiration, the lesion ball-valved between the supraglottis and subglottis, resulting in intermittent, near-complete glottic obstruction.

**Figure 1:**
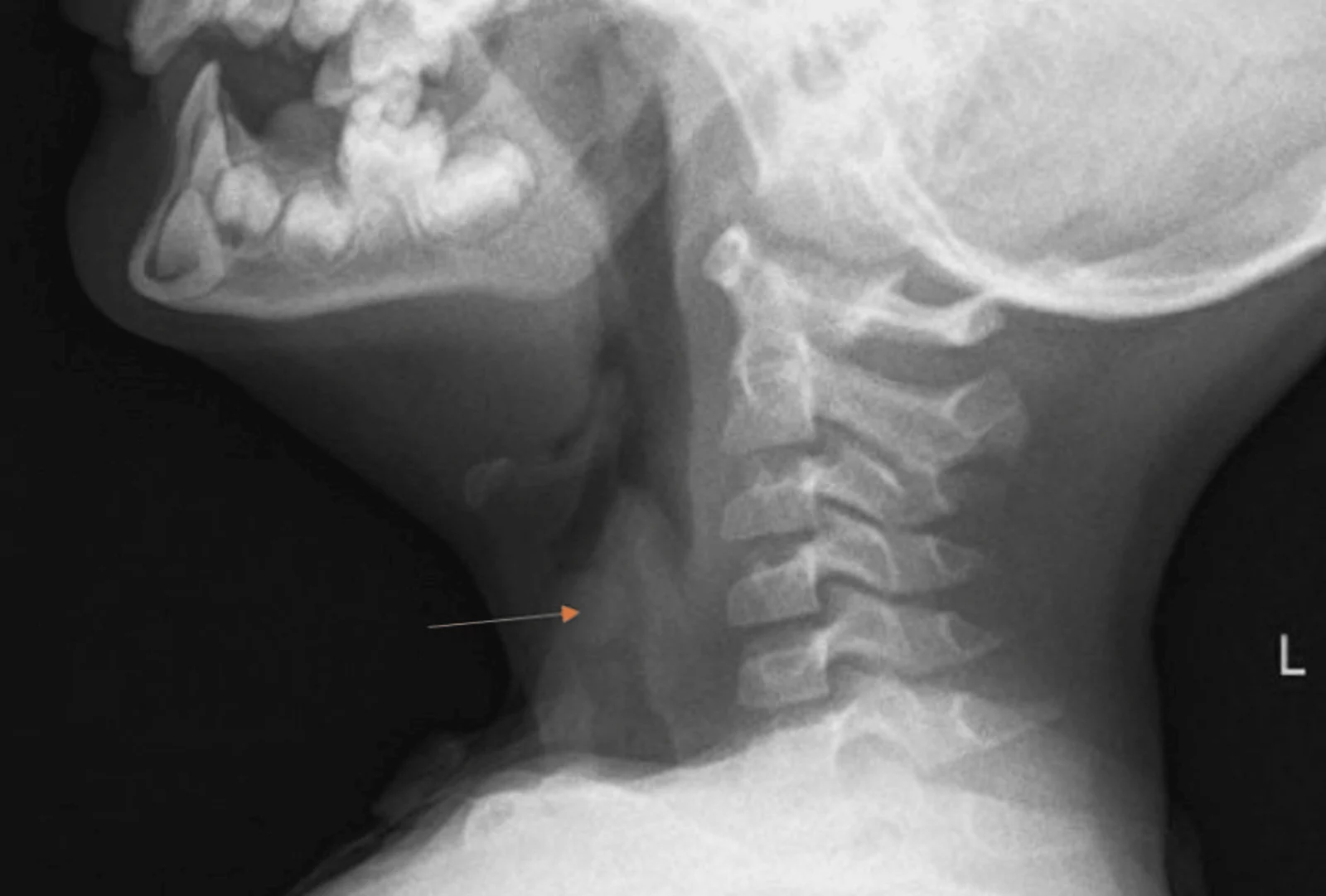
Lateral cervical X-ray showing soft tissue mass within the trachea.

**Figure 2:**
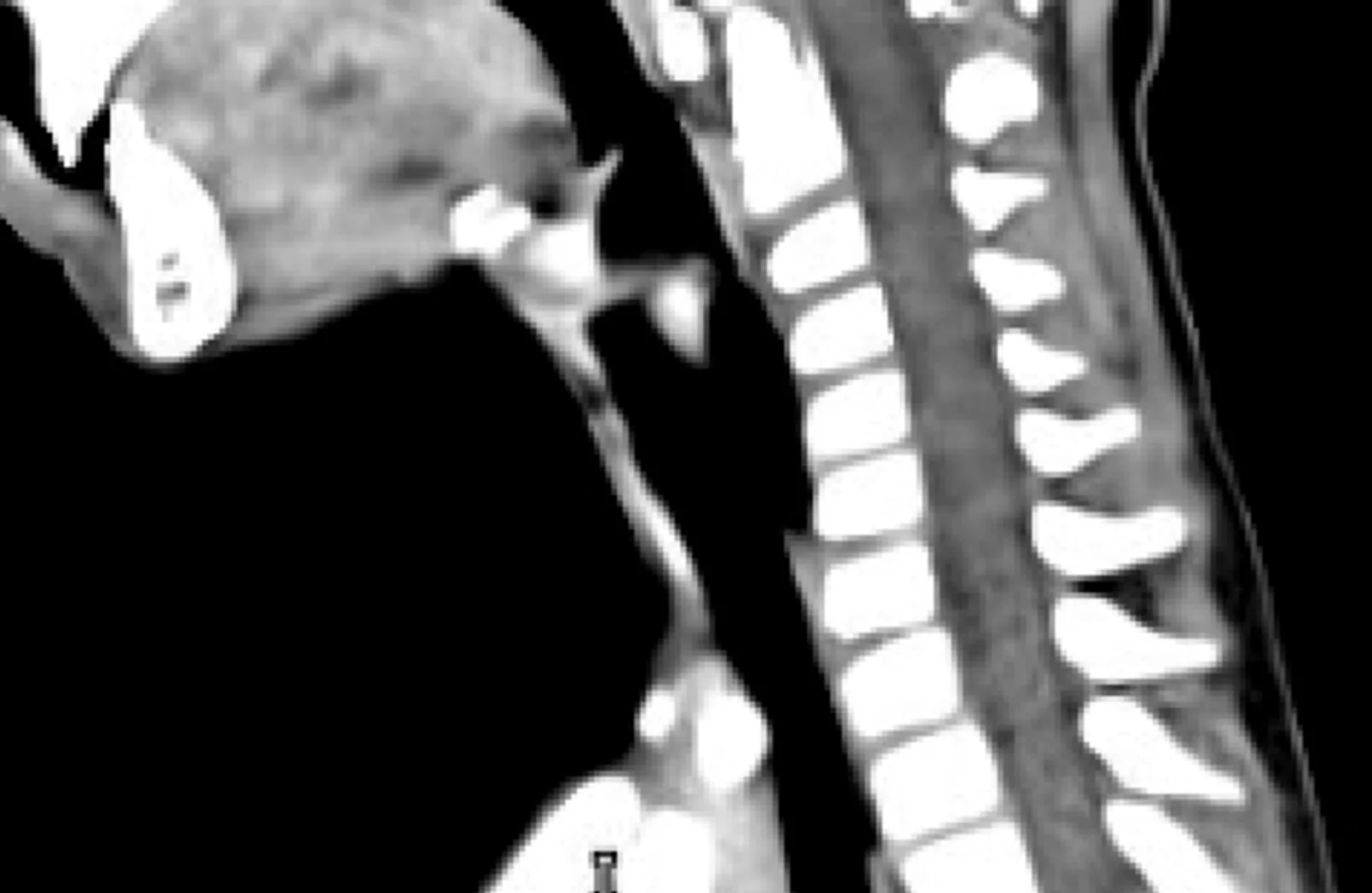
Sagittal CT with contrast that does not show an obvious obstructive mass in the glottis.

The patient was urgently taken to the operating room for direct laryngoscopy, bronchoscopy, and excision of the lesion. On direct laryngoscopy, the lesion had a narrow base and originated from the right mid-vocal fold (Figures 3, 4). Bronchoscopy revealed a clear subglottis and trachea. The patient was easily intubated with a 4.0 endotracheal tube. The laryngoscope was placed in suspension, and the lesion was removed in a piecemeal fashion under direct vision, with complete resection achieved (Figure 5). The patient was brought intubated to the pediatric ICU for monitoring due to extensive airway manipulation and the potential for postoperative glottic edema.

**Figure 3:**
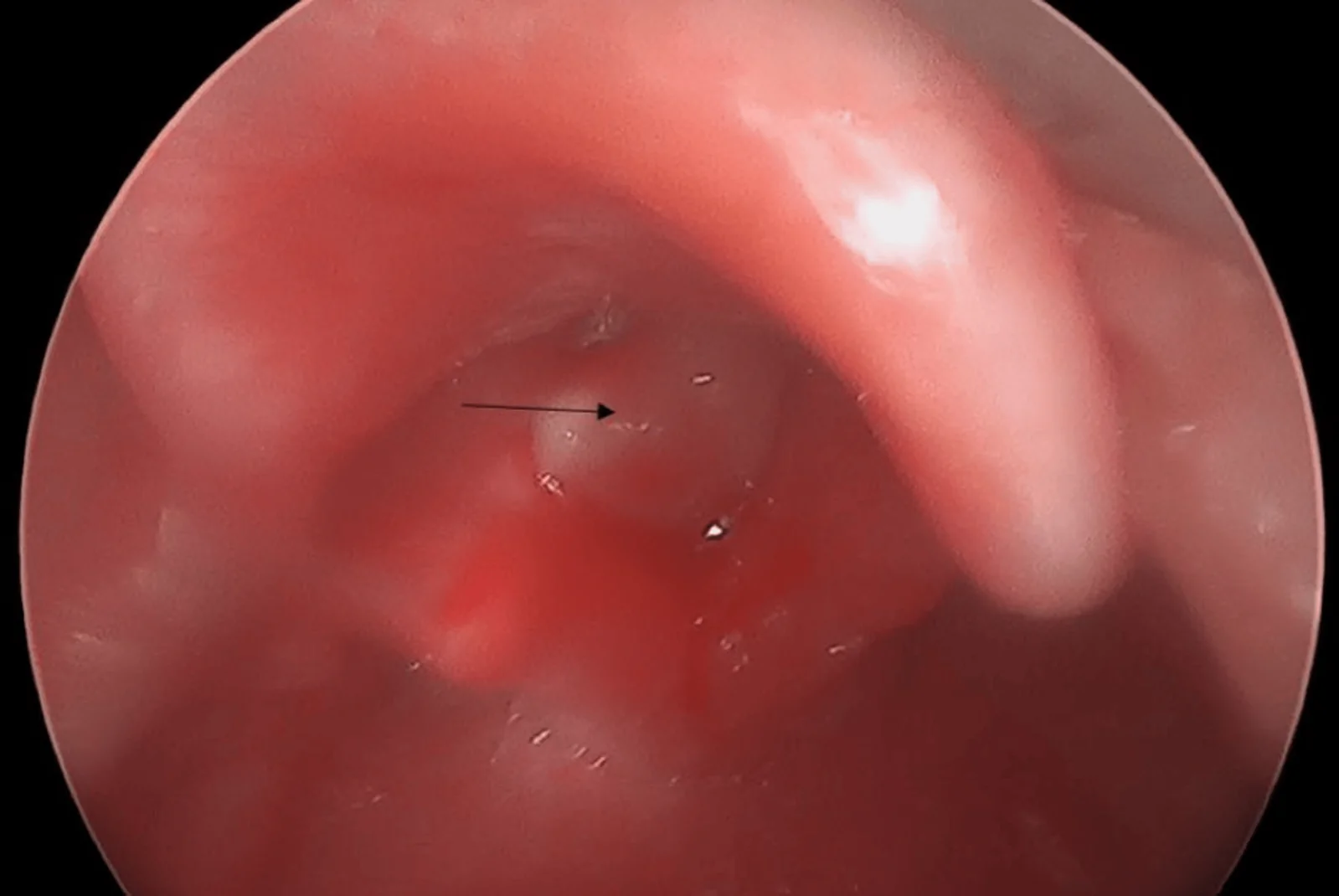
Laryngoscopy showing a soft tissue mass (black arrow) completely obstructing the glottis.

**Figure 4:**
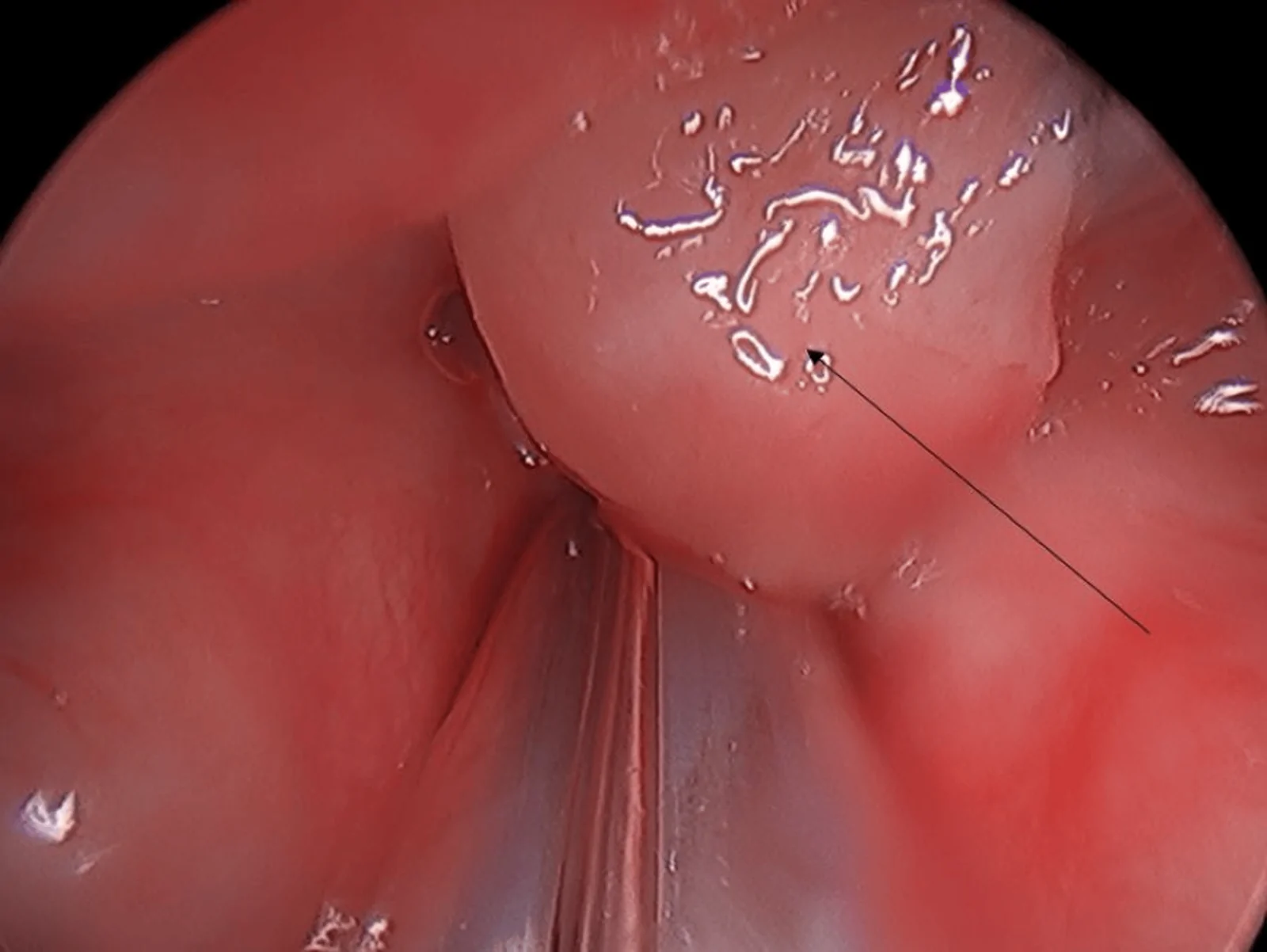
Direct laryngoscopic view of a pedunculated lesion originating from the right vocal fold.

**Figure 5:**
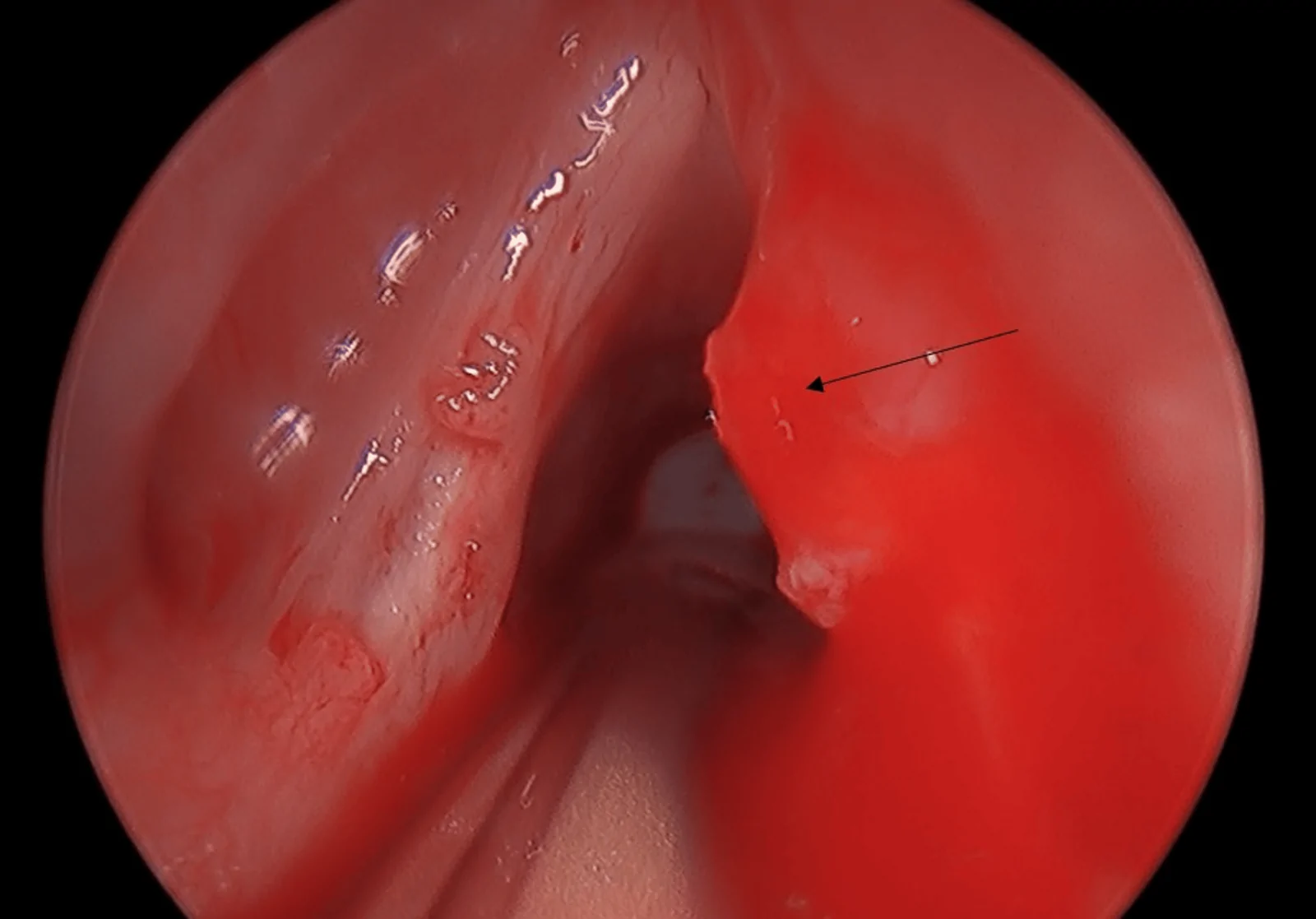
Immediate post-operative view following endoscopic excision of the right vocal fold mass. Complete resection was achieved and the glottic airway remained patent.

Postoperatively, the patient was treated with a course of intravenous steroids, and he was successfully extubated the next day with improved voice quality and resolution of stridor. At the patient’s six-week postoperative appointment, he had normal phonation with resolution of his hoarseness and no dyspnea, dysphonia, or stridor. The pathology was reported as a polypoid vocal fold granuloma with granulation tissue containing numerous small capillaries, fibroblasts, and an inflammatory infiltrate, covered with hyperplastic squamous epithelium. 

## Discussion

Pediatric VFG is uncommon. Consequently, the diagnosis can be easily overlooked when a child presents with stridor and voice changes, especially after a routine procedure. Intubation-related laryngeal injury in children spans a broad spectrum, from mild edema to granulation tissue formation and airway stenosis, with symptoms frequently not appearing immediately after extubation [3]. In this case, the child exhibited intermittent symptoms, and the initial workup was not straightforward. The mobility of the lesion likely explains both the fluctuating symptoms and the mismatch between the lateral neck radiograph and CT findings. This is also what made the lesion clinically dangerous. A pedunculated granuloma can create a true ball-valve lesion, in which the “ball” part of the lesion intermittently prolapses into the glottis, causing obstruction during portions of the respiratory cycle, offering a plausible explanation for this patient’s biphasic stridor [7,8]. This dynamic behavior renders the lesion particularly dangerous as it produces fluctuating but potentially severe airway compromise.

Although it cannot be definitively determined, the most likely precipitating factor in this case was the patient’s history of two brief intubations within the past six months. Prolonged intubation is a well-established risk factor for pediatric laryngeal injury. However, granuloma formation has been reported even after relatively short or routine airway instrumentation, which is important for interpreting this case [6]. Our patient’s history suggests that brief intubation does not eliminate risk, especially when there are multiple instances of airway instrumentation. Although it is impossible to determine whether a single intubation or the cumulative effect of both was responsible for the VFG, repeated instrumentation likely increased the chance of mucosal trauma and subsequent abnormal healing. Additionally, this susceptibility is anatomically plausible, as the medial aspect of the arytenoid is covered by a relatively thin layer of soft tissue and therefore is more vulnerable to pressure-related mucosal injury during intubation [1].

Management of VFG depends on both symptom severity and the degree of airway compromise. For nonobstructive lesions, conservative treatment is often attempted first and may include anti-reflux therapy, voice therapy, inhaled steroids, and observation [9,11]. In this case, the patient’s biphasic stridor and intermittent airway obstruction necessitated urgent operative management, not only to remove the lesion and confirm the diagnosis, but primarily to secure the airway. Direct laryngoscopy and bronchoscopy were particularly useful because they confirmed that the lesion arose from the right mid-vocal fold and allowed for removal during the same procedure. Piecemeal excision is also reasonable given the size and obstructive nature of the mass. More broadly, this case highlights that pediatric granulomas cannot always be managed like adult vocal fold lesions, especially when the primary concern is airway risk rather than isolated dysphonia [9,11].

This patient’s postoperative course is also worth discussing. The patient was kept intubated overnight in the PICU because of concern for glottic edema after substantial airway manipulation. Intravenous steroids were given before successful extubation the following day. Corticosteroids are commonly used as supportive treatment for their anti-inflammatory and edema-reducing properties when there are concerns about post-extubation upper airway edema. A systematic review and meta-analysis by Kimura et al. found lower odds of post-extubation stridor and extubation failure with corticosteroid use in children, making their use in this setting reasonable, although the literature was not specific to VFG [12]. The review included studies of extubation in cases of known or high-risk laryngeal edema and reported a range of different regimens of corticosteroid dosing, frequency, and timing in the trials studied [12]. In our patient, the short-term outcome was favorable, with successful extubation, improved voice quality, and resolution of stridor.

## Conclusions

This case highlights a critical and rare presentation of a ball-valving VFG in a child following repeat, brief intubations. VFG should be included in the differential diagnosis for pediatric patients with a history of intubation presenting with stridor and airway compromise, emphasizing that even short intubations can result in this complication.
